# Functional data analysis of sleeping energy expenditure

**DOI:** 10.1371/journal.pone.0177286

**Published:** 2017-05-10

**Authors:** Jong Soo Lee, Issa F. Zakeri, Nancy F. Butte

**Affiliations:** 1 Department of Mathematical Sciences, University of Massachusetts Lowell, Massachusetts, United States of America; 2 Department of Epidemiology and Biostatistics, Drexel University, Philadelphia, Pennsylvania, United States of America; 3 USDA/ARS Children’s Nutrition Research Center, Department of Pediatrics, Baylor College of Medicine, Houston, Texas, United States of America; Vanderbilt University, UNITED STATES

## Abstract

Adequate sleep is crucial during childhood for metabolic health, and physical and cognitive development. Inadequate sleep can disrupt metabolic homeostasis and alter sleeping energy expenditure (SEE). Functional data analysis methods were applied to SEE data to elucidate the population structure of SEE and to discriminate SEE between obese and non-obese children. Minute-by-minute SEE in 109 children, ages 5–18, was measured in room respiration calorimeters. A smoothing spline method was applied to the calorimetric data to extract the true smoothing function for each subject. Functional principal component analysis was used to capture the important modes of variation of the functional data and to identify differences in SEE patterns. Combinations of functional principal component analysis and classifier algorithm were used to classify SEE. Smoothing effectively removed instrumentation noise inherent in the room calorimeter data, providing more accurate data for analysis of the dynamics of SEE. SEE exhibited declining but subtly undulating patterns throughout the night. Mean SEE was markedly higher in obese than non-obese children, as expected due to their greater body mass. SEE was higher among the obese than non-obese children (p<0.01); however, the weight-adjusted mean SEE was not statistically different (p>0.1, after post hoc testing). Functional principal component scores for the first two components explained 77.8% of the variance in SEE and also differed between groups (p = 0.037). Logistic regression, support vector machine or random forest classification methods were able to distinguish weight-adjusted SEE between obese and non-obese participants with good classification rates (62–64%). Our results implicate other factors, yet to be uncovered, that affect the weight-adjusted SEE of obese and non-obese children. Functional data analysis revealed differences in the structure of SEE between obese and non-obese children that may contribute to disruption of metabolic homeostasis.

## Introduction

Adequate sleep is crucial during childhood and adolescence for metabolic health, and physical and cognitive development [1]. Inadequate sleep may be a risk factor for obesity, insulin resistance, diabetes, and cardiovascular disease in both children and adults [2]. Recent meta-analyses of cross-sectional and longitudinal studies confirm that inadequate sleep is a contributing factor for weight gain and the increased prevalence of childhood obesity [1–3]. Cross-sectional studies found that short sleep duration was associated with obesity [4–5]. Longitudinal studies confirmed the inverse association between sleep duration and body mass index (BMI) and weight gain [6–7].

Inadequate sleep induces neuroendocrine alterations that can cause an imbalance between food intake and energy expenditure leading to weight gain. Mechanisms for weight gain associated with inadequate sleep include increased food intake, shift in food preferences, reduced fat oxidation, reduction in physical activity, alterations in appetite-related hormones leptin and ghrelin, hedonic pathways, and extended time for ingestion [8]. In humans, insufficient sleep duration can disrupt circadian rhythms that are generated by central and peripheral clocks to maintain metabolic homeostasis [9]. Sleep restriction can affect the components of daily total energy expenditure including sleeping energy expenditure (SEE), resting metabolic rate, thermic effect of food and physical activity. Compensatory responses that increase energy intake and conserve energy expenditure may ensue in response to inadequate sleep. Indeed, sleep restriction studies have demonstrated decreased rates of energy expenditure [10].

Calorimeters are used to measure the energy expended or heat lost from the body [11]. Thermogenesis is a tightly controlled process within the body, but is influenced by individual factors such as age, sex, body size and composition, health and fitness, as well as ambient factors such as temperature and humidity. Experiments are designed to understand differences in energy expenditure between individuals, accounting for known individual characteristics and minimizing ambient influences. Despite such controlled experimental conditions, calorimeter data are subject to instrumentation noise, especially for low levels of energy expenditure during sleep [12–14]. Room respiration calorimeters are small rooms designed to measure respiratory exchange in subjects comfortably for relatively extended periods of time. SEE, measured by room calorimetry, is an excellent biomarker of an individual’s energy metabolism reflecting the energy needed to sustain the metabolic activities of cells and tissues, plus the energy to maintain blood circulation and respiration, minimally influenced by physical activity and consciousness.

However, there exist some challenges to analyzing SEE data. The SEE measurement generates data in continuous time with high correlation between measurements. In addition, data contain more time points than subjects, which makes the data high dimensional. Hence, the traditional multivariate statistical methods are not appropriate. To address this problem, we implement a specialized method, functional data analysis (FDA) [15], to analyze the SEE data in this study. An advantage of FDA is that it treats the whole measured function as a single entity, which is particularly useful to study the variation in the data. Specifically, FDA concerns the inference of data in which each observation is a function–a curve, surface, or anything else varying over a given domain, as opposed to a finite dimensional vector. The domain is typically a time, but also could be spatial location, wavelength, etc. These types of data—which usually consist of a large number of repeated measurements per subject—are collected in many fields of research, including physiological experiments which measure continuous energy expenditure.

The first step in FDA is signal extraction or statistical smoothing [16–18]. While the design of the room respiration calorimeters has taken noise reduction into account, there still exists some instrumentation noise from the system. Although the output may be acceptable under certain circumstances, the noise may obscure distinct patterns between individuals during sleep. Furthermore, the measured SEE by the room calorimeters is a realization of continuous physiological phenomena that are observed in discrete time. While the measured SEE time series are not often smooth but fluctuating, one can assume that the true underlying trajectory is a smooth function. Therefore, a more accurate and effective way to study the dynamics of SEE is to incorporate the information that is inherent in time order and smoothness of SEE series over time. For this reason, we applied a statistical smoothing method to calorimeter-generated data.

Moreover, we introduce functional principal component analysis (FPCA) [19] to make further inference about sleeping metabolism and to study the variability of SEE. Multivariate principal component analysis (PCA) is a well-known dimension reduction method widely known in many scientific communities [20]. FPCA is an extension of PCA that incorporates functional data, where FPCA not only performs dimension reduction but also gives graphical representation of the structure of data that reveals more about the data variability [15, 20]. FPCA techniques have been applied to diverse areas [21–23]. In FPCA, one explores the pattern of the results to interpret their physiological significance and to use it in conjunction with the algorithm for classification purposes. Although many studies have been published using the SEE data, such as prediction using time series and multivariate adaptive regression splines [24–25], the current work presents the application of FPCA on the SEE data for the first time.

Finally, we apply various algorithms (classifiers) to discriminate the SEE of obese and non-obese children. Many classifiers produce satisfactory results, but some classifiers are restrictive in that they do not allow full data as inputs. Applying the FPCA techniques in the classifiers reduces the number of inputs with the features extracted from FPCA and improves the classification rates versus using the full input. Furthermore, this technique enables a user to implement a simple classification tool such as logistic regression.

Given the shorter sleep duration reported in obese children and its potential to disrupt metabolic homeostasis, we apply advanced statistical methods to uncover differences in SEE between obese and non-obese children. The specific aims of this investigation are: 1) to apply signal extraction/statistical smoothing to SEE data; 2) to use FPCA to explore patterns and variability of SEE in obese and non-obese children; and 3) to implement FPCA and the classifier algorithms to uncover differences in SEE between obese and non-obese children.

## Materials and methods

### Study design and participants

The study is undertaken at the Children’s Nutrition Research Center (CNRC) of Baylor College of Medicine in Houston, Texas. The cross-sectional study design entailed measurement of minute-by-minute EE in 109 children and adolescents while inside a room respiration calorimeter for 24 hours [24–25]. Inclusion criteria stipulated that the children had to be 5 to 18 years of age, healthy, and free from any chronic diseases including sleep apnea, prescription drugs and medical conditions that would limit participation in physical activity or exercise. This study was designed and conducted in compliance with the principles expressed in the Declaration of Helsinki for research involving human subjects. The Institutional Review Board for Human Subject Research for Baylor College of Medicine and Affiliated Hospitals approved the protocol (Protocol Approval Number: H-12067, N. Butte, PI). All parents and children gave written informed consent to participate in this study.

Hispanic, African American, and Caucasian children, mean age 12.3 ± 3.5 years participated in the study. There were 46 girls and 63 boys. Table 1 displays the descriptive statistics of the 109 participants. Fifty-seven percent of the sample was classified as non-obese and forty-three percent as obese, defined as having a BMI ≥ 95^th^ Center for Disease Control and Prevention percentile [26]. Among the 46 girls, 20 were obese and 26 were non-obese, and among the 63 boys, 27 were obese and 36 were non-obese.

**Table 1:** Summary statistics of obese and non-obese participants (mean ± SD).

	Obese	Non-Obese	Total
n	47	62	109
Age (y)	12.3±3.3	12.3±3.6	12.3±3.5
Weight (kg)	73.1±27.0	45.0±16.0	56.6±25.3
Height (cm)	151.9±15.9	150.0±18.9	150.8±17.7
Raw SEE (kcal/min)	1.047±0.250	0.861±0.226	0.938±0.242
Weight-Adjusted SEE (kcal/min)	0.125±0.017	0.130±0.020	0.128±0.019

### Room respiration calorimetry

The CNRC room respiration calorimeters were used to estimate energy expenditure of children and adolescents during sleep from measured respiratory exchange of carbon dioxide for oxygen. Room-sized calorimeters were designed for the collection of respiratory gases from study participants in a comfortable, nonintrusive manner, but required high sensitivity and accuracy in instrumentation due to the large dilution of respiratory gases in the room. The design and performance of the room respiration calorimeters were presented in detail elsewhere [27]. Briefly, oxygen consumption ($\dot{V}$O_2_) and carbon dioxide production ($\dot{V}$CO_2_) are measured continuously in one of two 30-m^3^-room calorimeters for 24-h. $\dot{V}$O_2_ and $\dot{V}$CO_2_ are measured with paramagnetic oxygen (Oxymat 6) and nondispersive infrared CO_2_ (Ultramat 6) gas analyzers (Siemens, Karlsruhe, Germany) and thermal-mass flow controllers (model 740 and 840, Sierra Instruments Inc., Monterrey, CA). A microprocessor controller (CMP 3244, Conviron Ltd. Winnipeg, Canada) provides control of temperature and humidity within the calorimeter. Errors from 24-hour infusions of nitrogen and CO_2_ were -0.34 ± 1.24% for $\dot{V}$O_2_, and 0.11 ± 0.98% for $\dot{V}$CO_2_ [27]. In addition, heart rate is recorded by telemetry in the room calorimeter (DS-3000, Fukuda Denshi. Tokyo, Japan) and physical activity is monitored by a Doppler microwave sensor (D9/50; Microwave Sensors, Ann Arbor, MI). The sampling interval is one minute, which represents the average of ten six-second intervals. EE is computed using the de Weir equation [28].

The 24-hour calorimeter protocol included a series of scheduled physical activities, free time, meals, and sleep time. Participants were asked to prepare for sleep at 9:30 PM (lights out at 10:00 PM) and were awakened at 7:00 AM. Sleep was verified by heart rate and activity monitoring. Visual inspection of the accelerometer counts and heart rate data was used to identify nighttime sleep times. A plot of activity counts and heart rate per minute for each child’s 24-hour period was inspected to identify the time of sleep onset and termination. Sleep onset was identified by inactivity (accelerometer counts usually zero) and a gradual decline in heart rate. Sleep termination was identified by an abrupt increase in activity and heart rate. In-between sleep onset and termination, there is a period of quiescence with occasional excursions due to body movement. If there was evidence of awakening, these interruptions were excluded from the analysis.

Although the children were instructed to go to bed (lights out) at 10:00 pm, the exact time of sleep onset was not controllable. On average, sleep onset occurred 86 ± 53 (range 4 to 289) minutes before midnight.

### Normalization of data (allometric model)

Since the interest in this study was to compare the SEE of obese and non-obese participants, it would make sense to normalize the SEE patterns individually according to weight or other significant factors. For this task, we used the allometric model described in [29]. We fitted a power function regression, regressing the mean SEE on different candidate variables (weight, height, age, sex). To account for the discreteness of sex, we fitted a model

$$SEE=exp(\alpha +\beta ∙sex)\cdot {weight}^{\beta_{1}}{\cdot height}^{\beta_{2}}{∙age}^{\beta_{3}}\cdot \epsilon$$

To make the model additive, we took the natural log (ln) of both the response and the predictors and fit the model, i.e.,

$$\ln\left(SEE\right)=\alpha +\beta ∙sex+\beta_{1}∙\ln\left(weight\right)+\beta_{2}\cdot \ln\left(height\right)+\beta_{3}∙\ln\left(age\right)+ln(\epsilon )$$

The coefficients of every term (except age) were significant. However, compared to the model that had only the weight as the predictor, this full model only increased the (adjusted) R-squared by 0.02 (0.87 vs. 0.89). This suggested that the model with only the weight variable would be sufficient for our problem, which agreed with the results of [29].

Hence, we fitted the simple allometric model with only weight as the predictor. After fitting the model, the coefficient of the weight variable was found to be 0.5. Thus, the adjustment is achieved by dividing the SEE for an individual by the weight^(0.5). In this way, the weight effect is removed from the data. Such data are called allometrically scaled.

### Noise reduction and smoothing

As the room volume was the coefficient for the rate of gas accumulation, any noise present in the derivative was amplified dramatically. At near steady state conditions, the contribution of the derivative term was mostly amplified noise. Fortunately, the time constants of large rooms represented quite effective low-pass filters so that the derivative noise was almost entirely random.

Nevertheless, the resulting output of SEE suggested that there still existed some noise. Thus, a signal extraction method was implemented. In particular, a statistical smoothing method was ideal for this problem, since not only would statistical smoothing work well to solve the current problem, but also it would better equip us with necessary components for further analysis as a part of the FDA [15]. Since our data are of the time-series form with correlation between times, it is appropriate to use FDA, and the smoothing is the first step in the process.

In FDA the data are represented by the (smooth) function *f*_*i*_(*x*), *i* = 1,…,*n*, so that the inference is made with the functions rather than numbers. The mathematical set up of the statistical smoothing starts with the model

$$y_{it}=f_{i}(x_{it})+\epsilon_{it},\;i=1,\ldots ,n,\;t=1,\ldots ,T$$ (1)

Here, *y*_*it*_ denotes the observed SEE (data) for the *i*^*th*^ participant at time t, *x*_*it*_ corresponds to the time points *x*_*it*_, the *f*_*i*_(*x*_*it*_) is a signal (unknown), and *ϵ*_*it*_ is the iid random noise (error, unknown) with *ϵ*_*it*_ ∼ *N*(0,*σ*^2^) and the unknown *σ*^2^. The model (Eq 1) may be written in the form

DATA=SIGNAL+NOISE.

The goal of the smoothing is to estimate the smooth function *f*_*i*_ that best describes the data *y*_*it*_ by minimizing the noise subject to certain smoothness conditions. There are many smoothing methods available, as mentioned in Tokuyama et al. [30].

We use the smoothing splines, which is one of the most common and well-developed tools (including most computer programs) for this purpose [31]. For the smoothing splines method, we solve for *f*_*i*_ that minimizes

$$\frac{1}{T}\sum_{t=1}^{T}(y_{it}-f_{i}(x_{it}){)}^{2}+\lambda_{i}\int_{x_{i1}}^{x_{iT}}(f_{i}^{\left(m\right)}(x_{i}){)}^{2}dx_{i}$$ (2)

where $f_{i}^{\left(m\right)}$ is the *m*^*th*^ derivative of *f*_*i*_. We denoted the solution ${\hat{f}}_{i}$, which we call the smoothing splines smoother of degree 2m-1. Typically, we would take m = 2, so that we would have a cubic smoothing splines smoother (degree = 3). Looking at the formula Eq (2), “the first term measures the closeness to the data, while the second term penalizes the curvature in the function, and *λ*_*i*_ establishes the tradeoff between the two” [32]. Hence, the smoothness of the solution ${\hat{f}}_{i}$ will be achieved by varying the smoothing parameter *λ*_*i*_. The solution ${\hat{f}}_{i}$ is called the cubic natural smoothing spline. The vital task of selecting a good smoothing parameter *λ*_*i*_ will be described in the next section.

### Smoothing parameter selection

The smoother ${\hat{f}}_{i}$ depends on *λ*_*i*_ (the smoothing parameter), which controls for the smoothness of ${\hat{f}}_{i}$ and must be estimated [33]. We employ the following method for our project. In the smoothing splines, the solution ${\hat{f}}_{i}$ may be represented with the cubic B-spline basis,

$${\hat{f}}_{i}(x_{it})=\sum_{k=1}^{K}{\hat{c}}_{ik}\phi_{k}(x_{it})$$ (3)

where K is the number of basis functions, the functions *ϕ*_*k*_ are the B-spline basis functions, and ${\hat{c}}_{ij}$ are the coefficients of the basis functions. The reason for using the B-spline is that it has been shown to have more stable numerical properties than methods employing other basis [31–32], and so the usage of the B-spline is recommended and is common in statistics applications. In fact, most computer programs calculate the smoothing splines ${\hat{f}}_{i}$ in this way.

Selecting K has the effect of smoothing and hence can be performed instead of selecting *λ*_*i*_ directly. However, even selecting the number of basis functions K in an automated way is a difficult and unsolved problem in theory [34]. Hence, we will determine K by “myopic algorithm” (as explained in [34], or S1 Appendix). We shall call this procedure a B-spline method.

### Statistical inference of mean functions

Once we smooth the raw SEE data, we may take ${\hat{f}}_{i}$, the estimated SEE signal for each participant as individual data and perform the statistical inferences, such as testing for the equality of SEE between obese and non-obese groups. To accomplish this, we may take the mean at each time point for each of the obese and non-obese group, resulting in a point-wise mean SEE function for each group. Then we can test for the difference of mean SEE functions between obese and non-obese groups.

Such testing requires a specialized method since we are dealing with two smooth mean functions, and hence need the techniques from the FDA. There are many testing methods available, and we applied the adaptive Neyman and thresholding test [35] and the functional F-test [36]. However, often in FDA different testing procedures may yield different result, so that it may be desirable to perform post hoc testing [37]. One such technique was developed in [38]. The central idea of the technique is to test for the difference in means at every given time point (e.g., t-test at every time point) and apply a specialized multiple comparison procedure to adjust the p-values. With this testing procedure, we can test for the mean difference at arbitrary time points and determine at which time points the differences occurred, if any. All p-values will be obtained by simulation (permutation methods). Please see [38] for technical details.

### Functional principal component analysis (FPCA)

FPCA is similar to the multivariate PCA, except that each input vector is a function (or a function represented by a high dimensional vector) rather than a vector of numbers. FPCA has the same goal as PCA in that it strives to infer about the variability of data using graphical techniques. Overall, the FPCA procedure is similar to that of PCA but the mathematics and the computation are more complicated. Since the dimensionality of function is much greater than that of a vector used in multivariate analysis (where p>n in FDA), working directly with PCA will not work.

To compute the functional principal component, we need to construct a covariance function $\hat{v}(s,t)=\frac{1}{n-1}\sum_{i=1}^{n}\left[{\hat{f}}_{i}(s)-\bar{f}(s)\right][{\hat{f}}_{i}(t)-\bar{f}(t)]$ where $\bar{f}(x)=\frac{1}{n}\sum_{i=1}^{n}{\hat{f}}_{i}(x)$, and compute *μ* and *ξ*(*s*) sequentially by maximizing the objective function $<\xi ,V\xi >=\iint \hat{v}(s,t)\xi (t)dt\;\xi (s)ds$ subject to constraints ∫ *ξ*_*h*_(*s*)*ξ*_*h*_(*s*) *ds* = 1 and ∫ *ξ*_*h*_(*s*)*ξ*_*l*_(*s*) *ds* = 0 for *h* ≠ *l*, and $\int \hat{v}(s,t)\;\xi (t)dt=\mu \xi (s)$. For the actual computation, we make use of the basis functions (Eq 3), which is explained in Section 8.4 of Ramsay and Silverman [15] and implemented in a software package in the companion book [39] (more details are given in the S1 Appendix). Then we obtain the main ingredients of FPCA, the eigenfunctions *ξ*_1_(*x*),…,*ξ*_*H*_(*x*) (also called the principal components or harmonics), and the corresponding eigenvalues *μ*_1_,….,*μ*_*H*_. This has the effect of maximizing the variance of the individual component *ξ*_*h*_(*x*) but yet will be orthogonal to the any other components that we determine. We will select a few principal components based on the eigenvalues to represent the most variation of the data, where this practice is sometimes called dimension reduction, which is prevalent in statistics and large data studies [32]. We will also rotate the components (Using VARIMAX rotation [39]) to obtain the better graphical interpretation. When we rotate the principal components, we transform the eigenfunctions to make them more interpretable and achieve better visualization but yet preserve the orthogonality properties.

With the smooth data and functional principal components, we may define the functional principal component (FPC) scores. The purpose of the FPC scores is to give us a numerical summary of the component for each data point.

$$z_{hi}=\int \xi_{h}(t)[{\hat{f}}_{i}(t)-\bar{f}(t)]dt$$ (4)

We can also consider the VARIMAX rotated FPC components and obtain the rotated component scores. The scores will be plotted to investigate if any differences exist between the obese and non-obese groups, which ultimately assist us in classification.

### Classification algorithms

Suppose now that we have smooth data for inference and that we have computed the FPCA for further analysis. Using these tools, we are interested in developing an algorithm that will distinguish (classify) the SEE of obese and non-obese participants. Many state-of-the-art classification algorithms have been proposed [32].

In this work, we consider logistic regression (Logistic) [40], support vector machine (SVM) [41], and random forest (RF) [42–45]. A brief description of these classifiers is in the S1 Appendix and the references therein.

For all of the classification methods above, we consider all of the SEE data as inputs, as well as the reduced inputs with the FPC scores. In this way, we reduce the input dimension while retaining data features (and may improve on the classification rates). We have also considered age, sex and other information as inputs.

To obtain the classification rates, we fit the models and predict the outcome using the data. However, since we do not have separate training and validation sets needed for the typical classification process, we employed the K-fold cross validation (CV) [32, Section 7.10]. Here the observations (participants) are randomly divided into K equal sized sets, and one set of the K sets is left out. The classifier model is fitted with the K-1 sets, and validated with the remaining (left out) set. We then repeat this for each of the K sets, and attain the K-fold CV classification rate as the mean of the K individual classification rates. We use K = 10 for our problem.

To further obtain the reliable classification estimate, we repeat the process M times (Monte Carlo runs). This is done because the partition of the K-fold is random so that each time it gives us a different result, and hence we get a better sense of the performance of the K-fold CV by repeating the procedure M times (and can obtain the distribution of the K-fold CV classification rates). This describes precisely the Monte Carlo simulation of the K-fold CV, and we set M = 1,000. We report the Monte Carlo mean of the K-fold CV classification rates, which gives us a statistically consistent estimator. The K-fold CV can be performed and programmed on all of the classifiers.

## Results

### Data

Of 109 participants, we removed three due to insufficient SEE data. Among the remaining participants, each SEE data contain time points of at least 405 minutes, so that we work with n = 106 participants, each with T = 405 time points (i.e, the first 405 minutes of sleep).

The primary focus is to study the SEE pattern among the participants. Particularly, we are interested in comparing the SEE patterns between obese and non-obese groups. There are 44 obese participants and 62 non-obese participants for the analysis.

### Smoothing and parameter selection results

**Fig 1:**
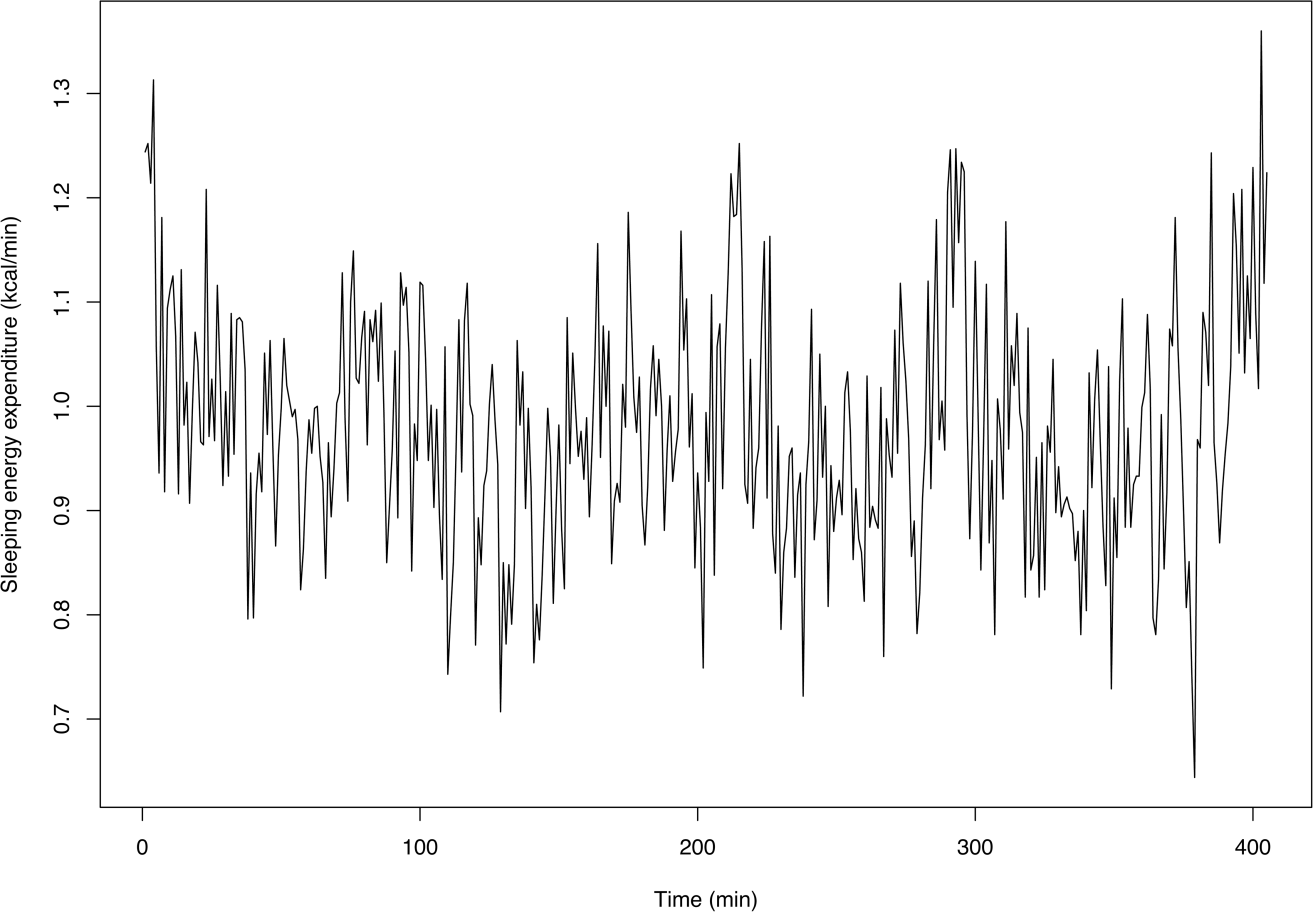
Raw sleeping energy expenditure (SEE) of an individual. A plot showing an example of raw sleeping energy expenditure (SEE) of an individual.

**Fig 2:**
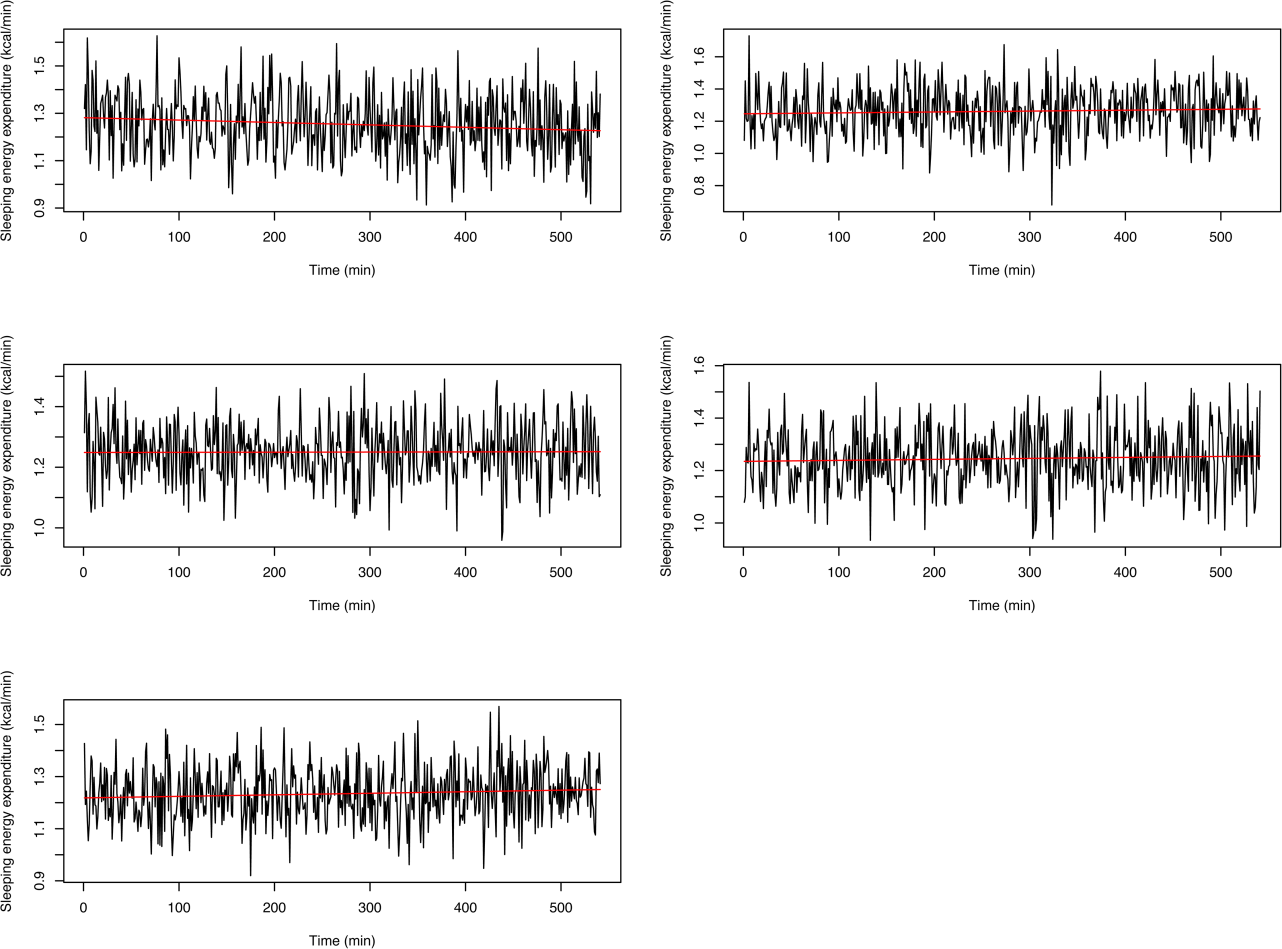
Constant infusions of N_2_ and CO_2_ into the room calorimeters to measure background noise. The measurements were taken five separate times. Red lines are smoothed infusion data, and the smoothing does not introduce artifacts.

An example of SEE output is given in Fig 1. The actual signals would be expected to be smoother than those represented in Fig 1. This has been confirmed by an analysis of constant infusions of N_2_ and CO_2_ into the room calorimeters to simulate human respiration. The room calorimeter measurement of the empty room was taken five times for 9 hours, and the results are shown in Fig 2. Clearly, system noise exists and hence smoothing is needed. The smoothing is also necessary for statistical inference purposes, since the smoothing removes the noise that may obscure one from performing the correct analysis (See, for example, [46]).

**Fig 3:**
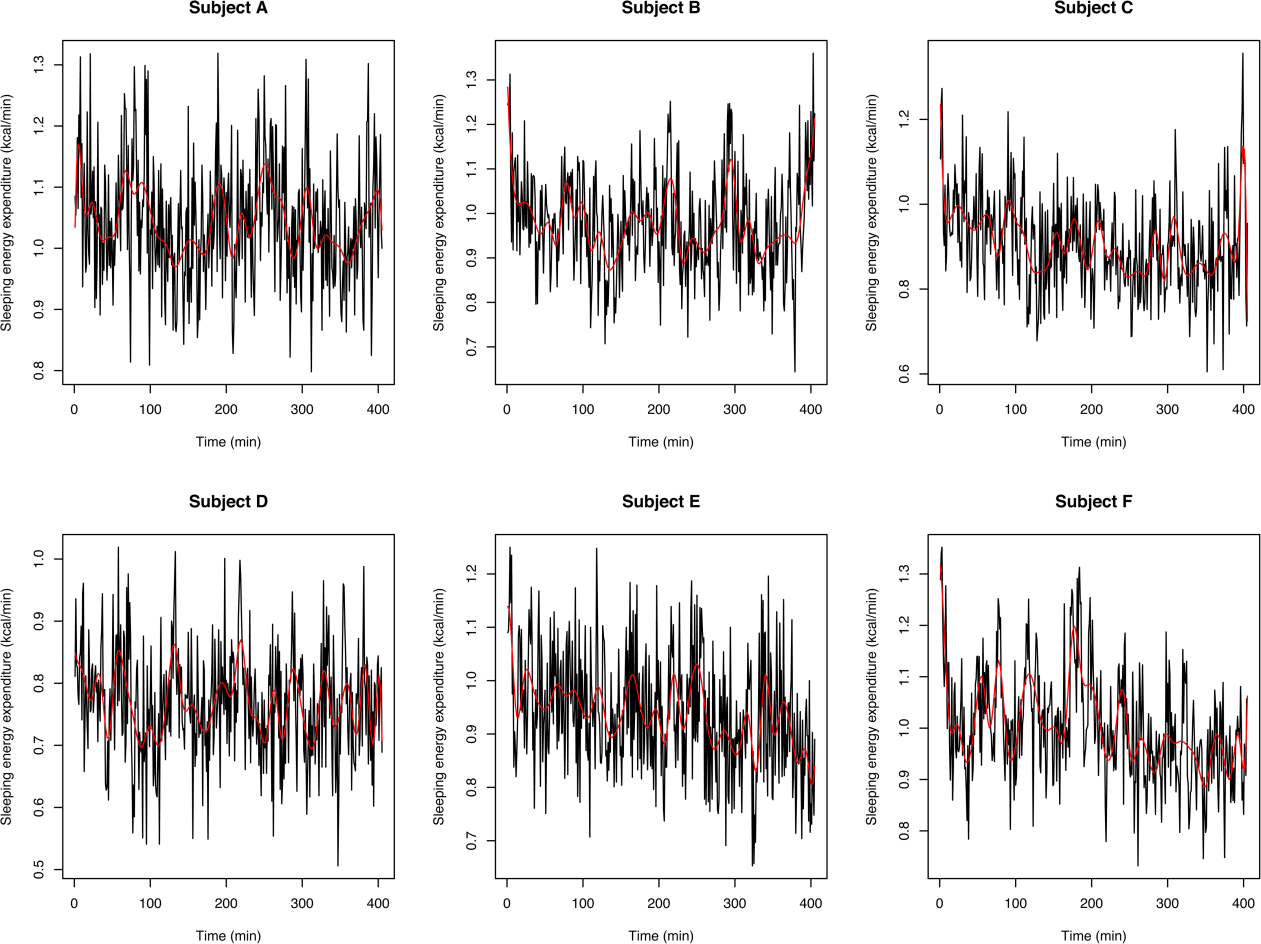
Smooth curves superimposed over raw energy expenditure (SEE). Plots showing raw SEE (black lines) along with the smoothed curves (red lines) using B-splines smoothing with K = 40.

For the smoothing and the smoothing parameter selection of the SEE data, we chose the smoothing splines smoother and the B-spline method (Recall Eq (3)). Based on the “myopic” algorithm in [34], we chose K = 40. The result gives us the right amount of smoothing for our data, as verified in Fig 3.

This method has generated curves that smoothed out most of the noise inherent in the data, giving a clearer picture of the SEE of the subjects.

### Comparison of mean functions

**Fig 4:**
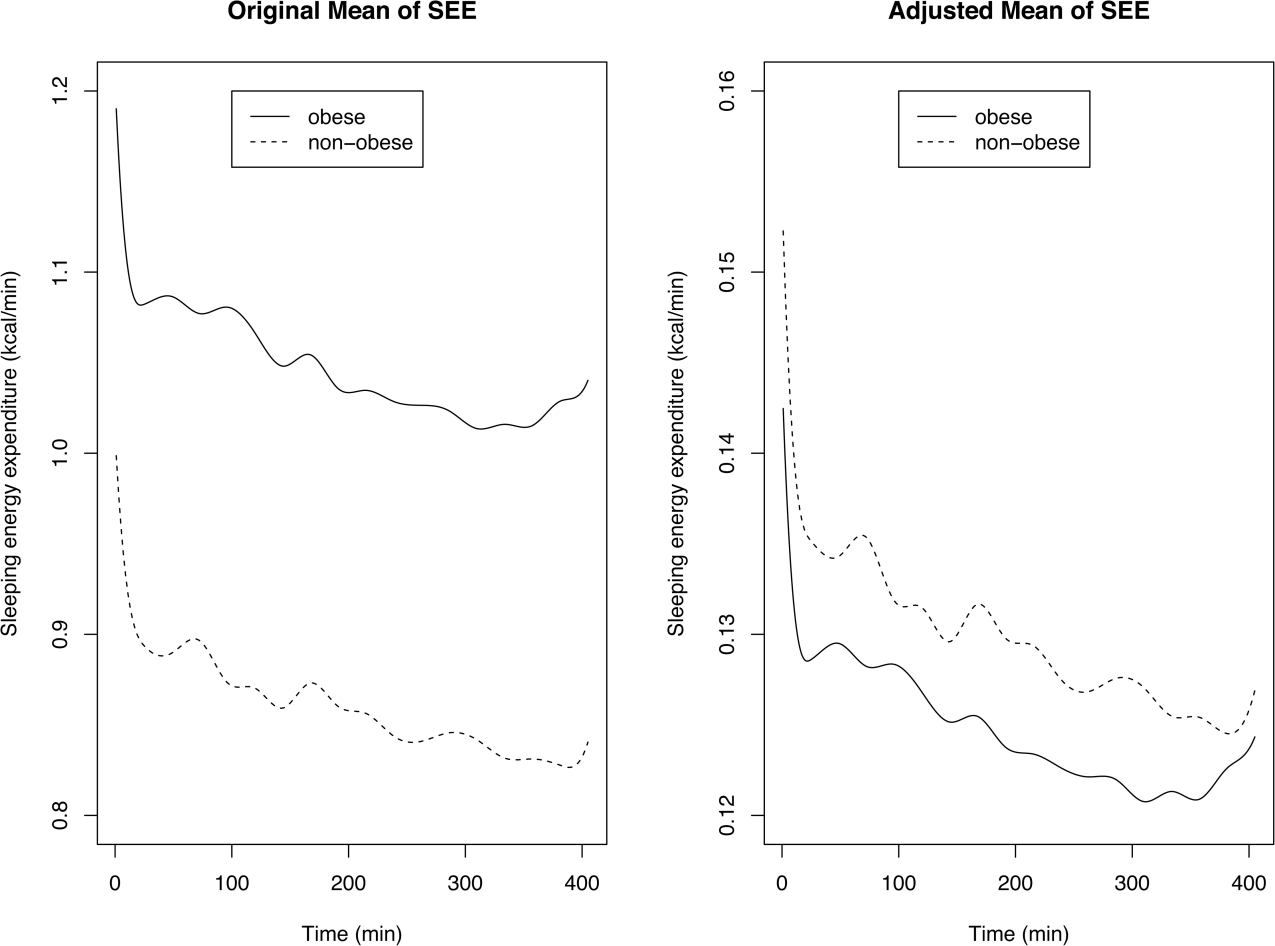
Comparing means of the sleeping energy expenditure (SEE). The left plot shows the mean SEEs at each time point. The right plot shows the means after adjusting data for weight.

For comparing the SEE patterns of obese and non-obese groups, it is natural to look at their point-wise mean functions. Fig 4 (left hand side) shows the point-wise means of obese and non-obese groups (without weight adjustment). SEE of the obese and non-obese children exhibited similar declining but subtly undulating patterns throughout the night, except at the beginning of sleep where a sharp drop is exhibited (as expected from the results of previous studies [47–49]). Mean SEE was markedly higher in obese than non-obese children, as expected due to their greater body mass. We see that both obese and non-obese groups exhibit very similar mean values over time but a shift between the two group means is apparent, with the obese group having a higher mean SEE than that of the non-obese group. However, the weight-adjusted data in Fig 4 (right hand side) show that the non-obese group mean is higher than that of the obese group, but the difference is noticeably smaller.

To formally test if differences exist between SEE of obese and non-obese groups in terms of their mean function, we employ the functional testing procedures introduced in the Materials and Methods section. As expected due to the differences in body mass, the difference between the two groups for the non-adjusted data was significant (p<0.01). For the weight-adjusted data, however, we obtain conflicting results with the adaptive Neyman test procedure [35] giving a non-significant result (p = 0.135) while the functional F-test procedure [36] giving a significant result (p = 0.024). To resolve this issue, we perform a post hoc testing procedure suggested in [38]. Here, we follow up the overall test procedure and see which variables (time points) are significant. The result is that at all time points, the adjusted p-values give non-significant results (p>0.1 at all time points). This means that at all individual time points, the differences are not significantly different. Hence, after normalizing for body mass, we claim that no differences in the mean pattern of SEE exist between obese and non-obese children.

From now on, we use the data that have been weight-adjusted. There was no difference whether adjusting the data first then smoothing or smoothing first then adjusting.

### FPCA results

For a deeper understanding of the patterns in our data that might have important implications, we implemented the FPCA. The advantage of FPCA (or PCA in general) is that we can select a few principal components to characterize the data, since only the first few components represent most of the variation in the data, which is of interest in our study. Hence, we need to decide how many components to keep for the FPCA. This is achieved by looking at the eigenvalues *μ*_1_,….,*μ*_*H*_ and their contribution to the overall variance, just like we would do in PCA. For the weight-adjusted data, the contribution to the total variance from the first component is 72.2%, and from the second component, the contribution is 5.6% or less. The first 8 components explain more than 90% of the total variability. Even though the contribution from the later components may be small, the combined components can together aid in visualization and classification.

**Fig 5:**
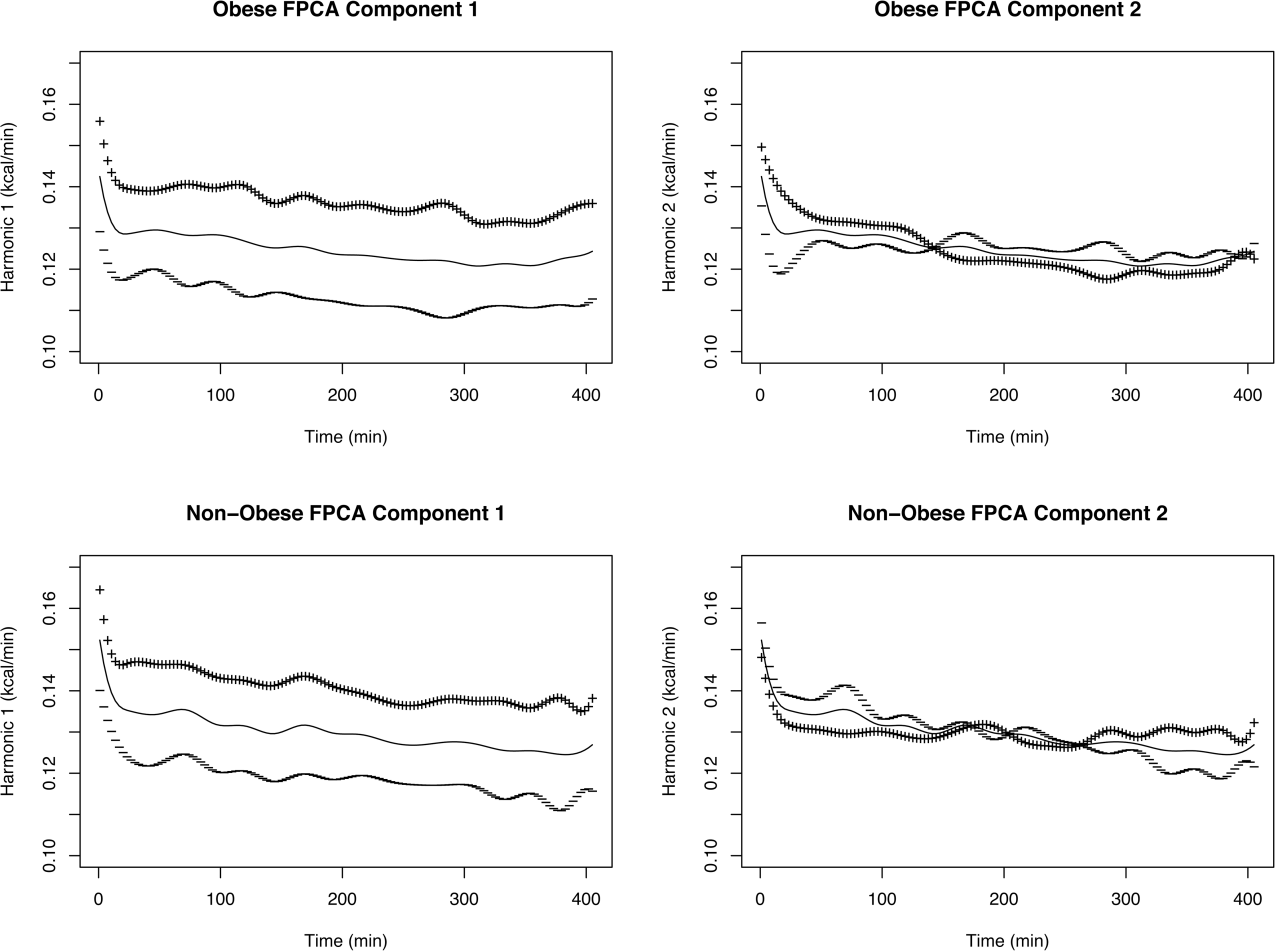
The functional principal component plots. The solid line is at the mean function, and the lines above and below are the functional principal component curves added (+) and subtracted (-) from the mean function. The first row shows the obese and the second row shows the non-obese components. The columns show the first and second FPCA components, respectively.

We provide several plots to help us interpret the results. First, we plot the FPCA components *ξ*_*h*_(*x*) to discern any difference in pattern between obese and non-obese groups. Fig 5 shows the plot implemented in the *fda* package in conjunction with FPCA. It plots the mean functions (solid lines) along with the square root of the eigenvalues times the components that were added (+) and subtracted (-) around the mean (i.e., $\bar{f}(x)\pm \sqrt{\mu_{h}}\times \xi_{h}(x)$) to better visualize the variations [39]. We observe that the first component of the obese group displays a slightly wider band than the non-obese group overall. This suggests that the obese group indeed exhibits more SEE variability than that of the non-obese group, since the first component corresponds to deviation from the overall mean. For the second component, we see now clearly different patterns for the two groups. The obese group again has more variability in the first 120 minutes in a different direction than the non-obese. The opposite phenomenon happens for later times (last 120 minutes, more variability for non-obese in a different direction). This indicates that the highest contributions to determining the SEE difference of the two groups come from the first and the last 120 minutes, which correspond to the periods of sleep onset and mid-sleep, respectively. Thus, the second component also distinguishes the SEE pattern. Together, both the first and the second FPCA components help determine statistically significant differences in SEE patterns. The distinct periods seen in the second component may correspond to REM/NREM patterns or subtle sleep disruptions. The origin of the two patterns of variability between obese and non-obese children is unclear; future EEG studies are required to explain the observed differences in sleep patterns between obese and non-obese children.

**Fig 6:**
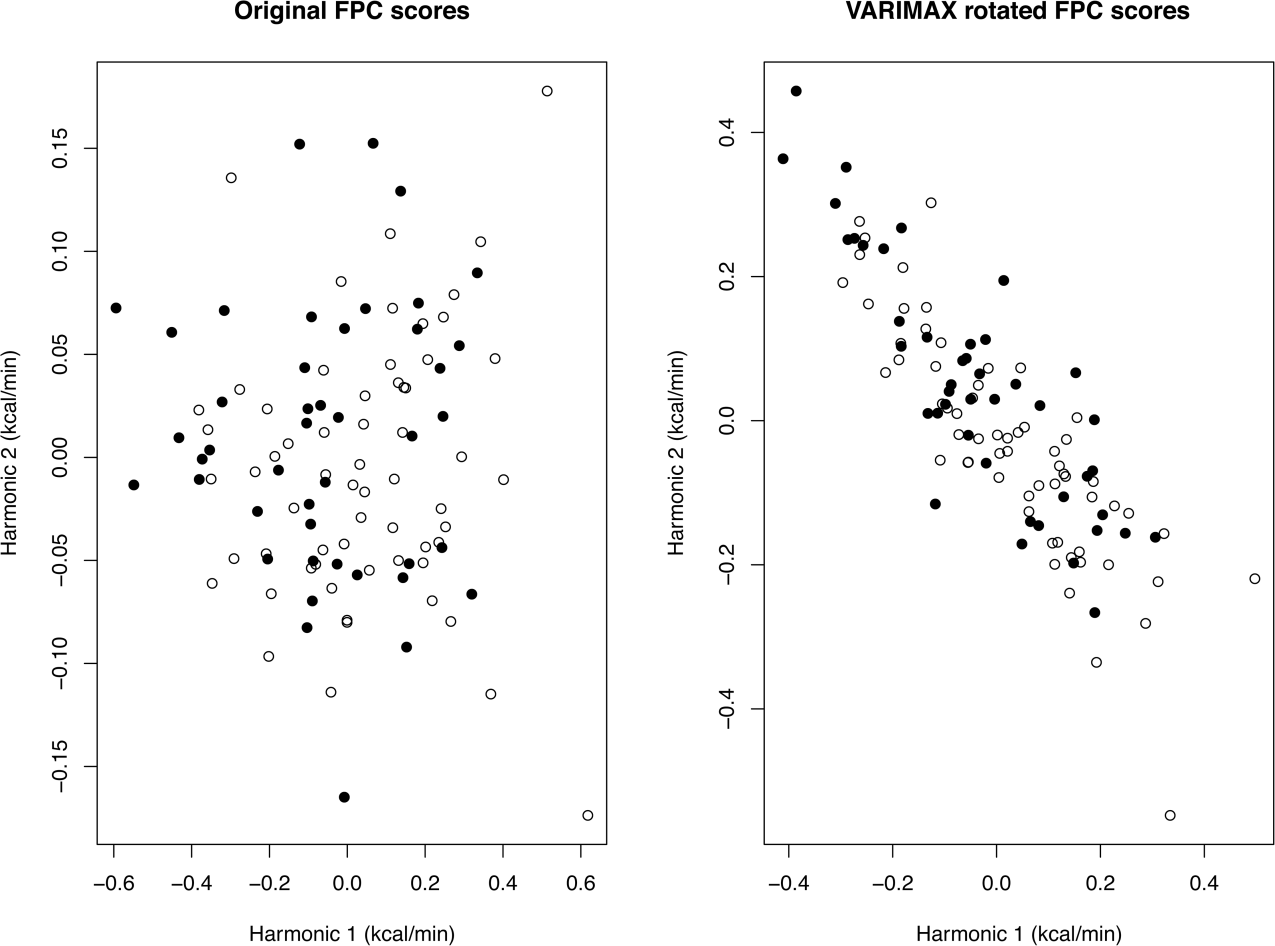
The functional principal component (FPC) scores for the individuals. The FPC scores are indicated by open circle (non-obese) or solid circle (obese). The plot on the left hand side shows the original FPC scores while the plot on the right hand side shows the VARIMAX rotated FPC scores.

We also plot the FPC scores *z*_*hi*_ (Eq 4) where h represents the components and i = 1,…,106 denote the participants. We can plot the points (FPC scores) (*z*_11_,*z*_21_),(*z*_12_,*z*_22_),(*z*_13_,*z*_23_),…,(*z*_1,106_,*z*_2,106_) so that we see each participant plotted on the x-y graph, where x-axis represents the first component and the y-axis represents the second component. For our problem, we have indicated the obese and non-obese group by plotting each point as either open circle (non-obese) or solid circle (obese) and observe if any pattern or separation emerges. See Fig 6 (left hand side). We also have the VARIMAX rotated scores plotted on the right hand side of Fig 6. As expected, the VARIMAX rotated scores give a better visualization of the patterns. The obese subjects tend to be clustered in the upper left corner, while the non-obese subjects are clustered in the lower right corner.

**Fig 7:**
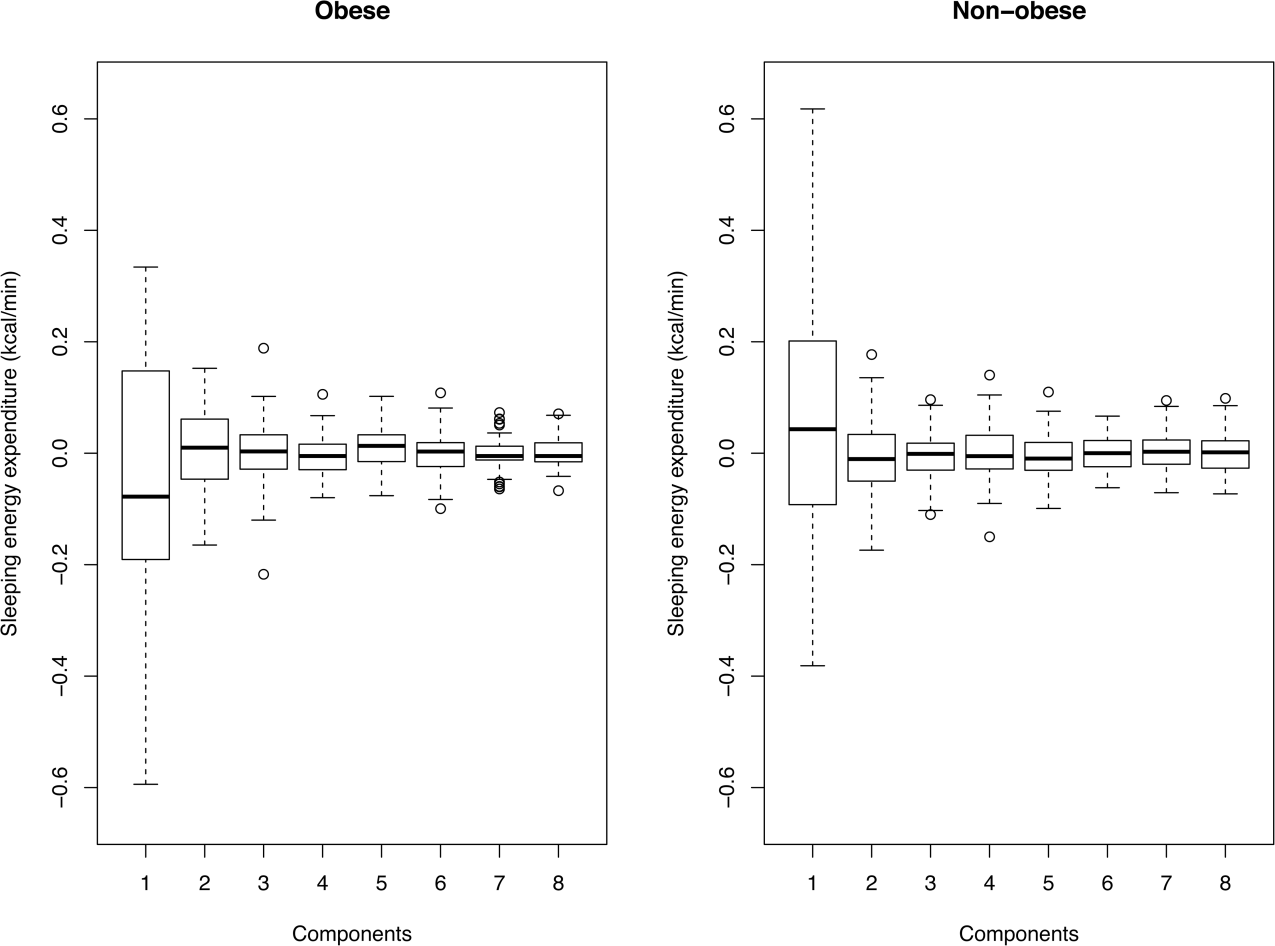
Boxplots of the first eight functional principal component (FPC) scores. The boxplots are plotted separately for obese and non-obese participants. The label in x-axis denotes the components (harmonics).

The summary statistics for FPC scores are provided in Table 2 and the boxplots of FPC scores are given in Fig 7. We see that the first component is the most variable in both obese and non-obese groups. We may test and see if the difference exists in FPC scores between obese and non-obese participants. To accomplish this, we fit a multivariate analysis of variance (MANOVA) model, with the FPC scores (2 or more components) as a response and compare obese and non-obese groups. As MANOVA with two groups is equivalent to the Hotelling’s T^2^ test [20], all tests give the same result. If we perform the test with 2 components, then we indeed see that the scores are significantly different between obese and non-obese groups (p = 0.037). However, the result is no longer significant if we have 3 or more components (p>0.05), which is expected because of higher dimension (multiple testing). Also, we have the same result if we use the VARIMAX rotated components, as expected.

**Table 2:** Summary of functional principal component (FPC) scores.

	Functional principal component scores (kcal/min)							
	1	2	3	4	5	6	7	8
Participants	Mean							
Obese	-0.060	0.010	0.003	-0.004	0.009	-0.001	-0.003	0.003
Non-Obese	0.043	-0.006	-0.002	0.003	-0.007	0.001	0.002	-0.002
	SD							
Obese	0.236	0.066	0.061	0.036	0.041	0.043	0.033	0.030
Non-Obese	0.223	0.063	0.044	0.047	0.041	0.031	0.035	0.036

Using FPCA alone we can discern the two groups, but it may not be definitive on its own. Using it in a combination with other techniques, such as classification algorithms, may give us more insights in understanding the distinction between the two groups.

### Classification results

As we have seen, it may be difficult to differentiate SEE between obese and non-obese individuals based on raw or smoothed weight-adjusted data alone. The basis expansion and FPCA seem to help in distinguishing the two groups, and we may want to investigate this phenomenon further with its effect on the classification.

Recall that we have three classification methods under consideration (Logistic, SVM, RF). For the data input, we try the full data (smoothed), and also the FPC scores with 2, 4 and 8 components. The VARIMAX rotated FPC scores are orthogonal transformation of the original FPC scores, so the results will be exactly the same.

We will perform the classification with the K-fold CV, as to avoid over-fitting of data and to enhance the predictive power. For the K-fold CV, we have attempted K = 10 as suggested by Hastie et al. [32]. Normally, a prediction error is presented as a result of 10-fold CV, but we are interested in the classification rate (which is simply one minus the prediction error rate).

Additionally, we repeat the process 1,000 times to give us the distribution of classification rates (or prediction error). For all the results, each number shown in the table is the mean of 1,000 Monte Carlo runs, which gives us a consistent estimator of the 10-fold CV classification rates.

The classification results are shown in Table 3. Note that some classifiers give better results with the full data, but the FPC scores with 4 components give the results as well as the full data. The lone exception was the logistic regression, which cannot be run with the full data, but performs relatively well if we input the FPC scores with 4 components.

**Table 3:** Classification rates (one minus the prediction error rates) of weight-adjusted SEE between obese and non-obese participants using full data or functional principal components (FPC).

Classification method	Full data	FPC 8	FPC 4	FPC 2
Logistic regression	NA	59%	63%	60%
Support vector machine	64%	62%	62%	59%
Random forest	62%	58%	62%	51%

The results also confirm that logistic regression is a very useful tool if we can use the reduced dimension data, as we did with the FPC scores. Because logistic regression is a well established and understood method, it is a practical method to use.

We considered adding some individual characteristics as features (age, sex and height) to see if they improve on classification. Addition of these covariates did not improve on the classification of the weight-adjusted SEE data.

REMARK: We have also attempted to apply a penalized variable selection/reduction method such as elastic net, where the least absolute shrinkage and selection operator (LASSO) is a special case, described in [50] and implemented by glmnet function in R. We obtain results comparable to that of FPC-based results. For example, we ran glmnet with LASSO and logistic regression, and obtained the classification rate of 63%. Other variations of glmnet give similar results. However, the interpretation become more difficult with glmnet, because it selects time variables at disparate times while our data has time variables that are correlated. Hence, it is more sensible to consider FPCA in our problem. The details are explained in the S1 Appendix.

In summary, we may use only the 4 components of FPC and still obtain a good classification rate of weight-adjusted SEE (62–64%). Simple classifiers like logistic regression on the 4 components can be used to derive the classification rate (63%) and can perform as well as other sophisticated algorithms.

## Discussion

In the pediatric literature, there are many publications on resting metabolic rate or basal metabolic rate, which is a 30–60 minute measurement performed in the awake state under standardized conditions, but there are few studies on sleeping energy expenditure [51–57]. While sleeping energy expenditure can be measured using a metabolic cart, room respiration calorimeters are preferable since they can nonintrusively measure sleep energy expenditure for extended periods of time. However, room calorimeters such as those located at the Children’s Nutrition Research Center are not widely available.

Using the Children’s Nutrition Research Center room calorimeters, we have evaluated energy expenditure and respiratory quotient (RQ) in other studies of children, including those with obesity [51]. Absolute total energy expenditure (EE) and its components (sleep EE, basal EE, sedentary EE, cycling EE, walking EE, activity EE, nonexercising activity thermogenesis) were higher in obese children (P = 0.001). Adjusting for body size and composition accounted for differences in TEE, its components, and energetic efficiency. Respiratory quotient, net carbohydrate and fat utilization did not differ between non-obese and obese children. Since there was no evidence of differences in substrate utilization between non-obese and obese children, we chose to focus on patterns of SEE in this analysis.

In this study, we have applied a statistical technique, FDA [15], to model and explore differences in the patterns of SEE measured by room respiration calorimetry between obese and non-obese children. To address the inherent instrument noise in calorimetric data [12–14], smoothing was necessary to extract the minute-to-minute SEE values during nighttime sleep. The smoothing splines smoother and B-spline method [32] were applied to the calorimeter data during 405 minutes of nighttime sleep. Statistical smoothing effectively removed instrumentation noise inherent in the room calorimeter data, providing more accurate data for analysis of the dynamics of SEE.

Once the smoothing was done, we compared the patterns and mean SEE between obese and non-obese groups. SEE exhibited similar declining but subtly undulating patterns throughout the night in both groups. Mean SEE was markedly higher in obese than non-obese children, as expected due to their greater body weight. SEE, adjusted for weight, tended to be higher in the non-obese, but the difference was smaller and nonsignificant. After normalizing for weight, the mean patterns of SEE were similar; however, the difference in variability of SEE was detectable by visualization and by considering FPC scores. The first two FPCA components explained 77.8% of the variance. VARIMAX rotation of the first two FPC scores gave a better visualization of the patterns with the obese subjects clustering in the upper left corner, and the non-obese subjects in the lower right corner. The FPCA of the first two components were the most variable and differed significantly between obese and non-obese groups. Furthermore, the exploration and analysis of the FPCA components revealed different patterns in SEE between obese and non-obese groups, possibly attributed to differences in REM/NREM sleep of subtle sleep disruptions. However, we would need more comprehensive studies, such as EEG study, to definitively prove the connections and better explain the results in terms of physiology.

Even after accounting for weight, classification methods (logistic regression, support vector machine or random forest) were able to distinguish the SEE between obese and non-obese participants [32]. Performing classification with the incorporation of FPCA further confirmed the statistical significant difference in SEE. All of the classification methods performed similarly, but we recommend the use of logistic regression because it is easily understood and implemented. Based on the four FPCA components, logistic regression provided good classification of weight-adjusted SEE (63%). Child characteristics such as age, sex and height did not improve on the classification of the weight-adjusted SEE data. Thus, our results imply that other factors, yet to be uncovered, affect the variability of SEE in obese and non-obese children. Neuroendocrine alterations and disruption of circadian rhythms associated with obesity may affect the variability in SEE [2, 8, 9].

In conclusion, FDA is a modern statistical technique that greatly aids in uncovering phenomenon in physiological systems. FDA revealed differences in the structure of SEE between obese and non-obese children that may be associated with disruption of metabolic homeostasis.

## Supporting information

S1 Appendix Appendix containing computer codes and mathematical details. (DOCX)

S1 Dataset Dataset used for this study. (CSV)
