# Supplementary material for: Functional data analysis of sleeping energy expenditure
Source: PLoS One. 2017 May 10;12(5):e0177286. doi: 10.1371/journal.pone.0177286 (PMC5425044; doi:10.1371/journal.pone.0177286)
Supplement: S1 Appendix — (DOCX) [file pone.0177286.s001.docx]

**S1 Appendix**

**A. Computing details and codes**

We use the statistics program R (R software, <https://www.r-project.org>) with the accompanying *fda* package [39]. Although there are many packages available on R to perform the analysis, the *fda* package is designed specifically for the analysis of functional data and unified different FDA methodologies in one package. This package allows us to perform state-of-the-art statistical analysis such as FPCA, but it can also perform the smoothing as well.

The smoothing is done in the *fda* package in R as follows. First, we create a B-spline basis with the number of knots K specified using create.bspline.basis() function, then smooth the data with K knots via smooth.basis() function and create the “functional data object” to be used for the FPCA analysis and graphics [39].

All of the classification methods are also available in R, although many require the use of specialized packages as described below. One exception is the logistic regression, which does not require any specialized package; this classifier only needs the built-in glm() function. For the SVM, we use *e1071* package with svm() function, with the default radial basis kernel and tuning parameter. Finally, we use *randomForest* package with the randomForest() function. For K-fold CV, even though some R classification functions include options to perform it, we decided to program our own simple K-fold CV function so that we have a fair comparison across all the classifiers.

As with many contributed packages in R, the packages we encountered were not straightforward to implement for certain applications of FDA and classification. We therefore illustrate how to use the package and provide the computer codes below.

Suppose that we have the individual SEE data (length T=405), and call it SEE. Fitting smoothing splines and finding the fit by GCV is achieved simply by using smooth.spline() with

> # run the smoothing splines in R, with data SEE.

> smooth.spline(SEE)

> # gives us the value of resulting fit, using GCV(default)

> SEE.GCV <- predict(smooth.spline(SEE))

For the GML fit, we can use a package *gss* and function ssanova(), as follows

> # load the gss package for computing GML

> library(gss)

> # here need to specify x values

> x <- seq(1,405)

> # let y be the data

> y <- SEE

> # fit the smoothing splines with GML, using ssanova() (method=”m” requests GML fit)

> gml.fit <- ssanova(y~x,method="m")

> # need a pre-step to give us predicted values

> new <- data.frame(x=seq(min(x),max(x),len=length(x)))

> # gives us the value of resulting fit, using GML

> SEE.GML <- predict(gml.fit,new)

To use the *fda* package to achieve the desired smoothing with B-spline, we need to adhere to the following sequence. In particular, we extract fd (functional data) component from bspline object (using first create.bspline.basis() then smooth.basis() functions) to be used in the subsequent analyses

> # load the fda package

> library(fda)

> # create cubic B-spline basis with desired number of knots (40)

> basisobj.40 <- create.bspline.basis(c(1,405), 40)

> # smooth the data using B-splines

> SEE.bspline <- smooth.basis(argvals=1:405, y=t(SEE), fdParobj=basisobj.40)

> # extract “functional data object” for use in analysis

> SEE.bspline.fd <- SEE.bspline$fd

> # gives us the value of resulting fit

> SEE.bspline.hat <- eval.fd(c(1:405), SEE.bspline.fd)

We can then compare the values SEE.GCV, SEE.GML and SEE.bspline.hat.

To plot the results, one can use the predicted values above or use the built-in functions of the libraries to plot the results directly (i.e., no need to use predict() or eval.fd() functions to extract fitted values in some cases). Here are excerpts of codes for drawing the plots (given that we have already run the above codes)

> plot(SEE, type='l', xlim=c(1,405), xlab='Time (min)',

ylab='Sleeping energy expenditure (kcal/min)')

> lines(SEE.bspline.fd, col='red',cex=1.5)

To perform the FPCA, we again use the *fda* package. In particular, we use pca.fd() for performing FPCA with the input from previous fd object, and we then use varmx.pca.fd() to perform VARIMAX rotation.

>SEE.bspline.pca <- pca.fd(SEE.bspline.fd, nharm=2)

>SEE.bspline.pca.varimax <- varmx.pca.fd(SEE.bspline.pca)

We may obtain FPC scores by simply taking the score component

>SEE.bspline.pca.score <-SEE.bspline.pca$scores

The VARIMAX rotated FPC scores may be obtained similarly

>SEE.bspline.pca.varimax.score <-SEE.bspline.pca.varimax$scores

To plot the FPCA components, we use the plot.pca.fd() function with an fd object as an input, for example,

>plot.pca.fd(SEE.bspline.pca)

One needs to modify the code to distinguish obese and non-obese plots and to display correct labels. For the FPC score plots, one simply uses plot() function with the scores

>plot(SEE.bspline.pca.score[,1], SEE.bspline.pca.score[,2])

>plot(SEE.bspline.pca.varimax.score[,1], SEE.bspline.pca.varimax.score[,2])

where we again need to add some additional options (particularly to identify the obese and non-obese subjects) in the plot function.

For classification, we use the following code

kfold.cv <- function(fn, data.input, k, M){

ind.class<-rep(NA, M)

for(i in 1:M){

data.sample<-data.input[sample(nrow(data.input)), ]

folds<-cut(seq(1,nrow(data.sample)), breaks=k, labels=FALSE)

class.valid<-rep(NA, k)

for(j in 1:k){

ind.valid<-which(folds==j,arr.ind=T)

data.train<-data.sample[-ind.valid, ]

data.valid<-data.sample[ind.valid, ]

fn.train<-fn(factor(y)~. , data=data.train)

fn.pred<-predict(fn.train,newdata=data.valid[ ,-1])

class.valid[j]<-mean(data.valid[ ,1] == fn.pred)

}

ind.class[i]<-mean(class.valid)

}

return(ind.class)

}

The inputs are

fn : The classification function used: glm, svm, randomForest

data.input: Must be in R data frame. The first column is the response (y), and the rest of the columns are predictors.

k: The number of k fold

M: The number of Monte Carlo simulations

As an example, if the SEE.FPC4 is the data with 4 FPCs from out data, the code

> kfold.cv(svm, SEE.FPC4, 5, 1000)

gives 1,000 Monte-Carlo, 5-fold CV classification for the SVM classifier.

**B. Additional Details of Smoothing and Parameter Selection**

In this section, we present the further details of smoothing splines and the mathematical details of the smoothing parameter selection.

The smoothing spline is a special case of the spline method in mathematics (in approximation theory) [31]. A spline function is a piecewise polynomial function that approximates a given function. The piecewise polynomial develops between knots, where the knots are located at time points (to begin with, since the number and location of knots may be enlarged). The most frequently used polynomial degree is the cubic, called the cubic spline. In practice, it is common to use a specialized spline called the natural cubic spline, mainly to combat the issues on the boundary [31]. In statistics, a variant of cubic spline, called the smoothing spline, is mostly employed. The details of spline functions, and their relationship with smoothing splines, are given in Hastie et al. [32, pages 139-153].

Here are the details of the smoothing parameter selection. The cross-validation (CV) as a function of $\lambda_{i}$ (the smoothing parameter) is defined as

$$CV\left( \lambda_{i} \right)=\frac{1}{T}\sum_{t=1}^{T} (y_{it}-\hat{f}_{i,\lambda_{i}}^{\left( t \right)}(x_{it}))^{2}$$

where $\hat{f}_{i,\lambda_{i}}^{\left( t \right)}$ is the estimator with smoothing parameter $\lambda_{i}$ and with the observation $\left( x_{it},y_{it} \right)$deleted [32]. One would choose $\lambda_{i}$ by minimizing $CV\left( \lambda_{i} \right)$.

For computational convenience, a more popular choice for the selection of $\lambda_{i}$ is by an approximation of CV, the generalized cross-validation (GCV) [16]. The GCV is defined as

$$GCV\left( \lambda_{i} \right)=\frac{(\frac{1}{T})\left\| [I-A\left( \lambda_{i} \right)]y_{i} \right\|}{{[\left( \frac{1}{T} \right)tr\left( \left[ I-A\left( \lambda_{i} \right) \right] \right)]}^{2}}$$

where $y_{i}= (y_{i1}, . . . , y_{iT} )'$ is a vector of SEE for individual i, tr(·) is the trace function, $\left\| \cdot\right\|$ is the euclidean norm, and $A(\lambda_{i})$ is the T×T smoother matrix satisfying

$$\left( \begin{matrix} \hat{f}_{i}\left( x_{i1} \right) \\ \vdots\\ \hat{f}_{i}\left( x_{iT} \right) \end{matrix} \right)=A(\lambda_{i})y_{i}$$

It can be shown that GCV approximates CV well and can be much faster to compute [16]. The fitting of the (cubic) smoothing splines with CV or GCV is discussed in literature and widely available in many programming packages, including the default smooth.spline() function in R.

However, in practice, GCV tends to undersmooth the functions when we desire a smooth curve. This was the case in our current project. Hence, it would be desirable to find an alternative method for selection of λ.

One of the alternatives we tried was the generalized maximum likelihood method (GML), which is not a well-known method by general audiences and has only appeared in the specialized smoothing splines literature [33]. Heuristically, the GML method utilizes the maximum likelihood (derived from the Bayesian idea of splines) to obtain the best smoothing parameter. The GLM is defined as

$$GML(\lambda_{i}) =\frac{y_{i}^{'}[I-A\left( \lambda_{i} \right)]y_{i}}{{({det}^{+}\left[ I-A\left( \lambda_{i} \right) \right])}^{1/(T-m)}}$$

where ${det}^{+}\left[ I-A\left( \lambda_{i} \right) \right]$ is the product of (T – m) nonzero eigenvalues of $[I - A(\lambda_{i})]$ [33]. Again, $\lambda_{i}$is chosen by minimizing $GML(\lambda_{i})$. The derivation of the GML function is given in [18, 33], and we omit the detail here. Contrast this with the CV method that involves “validating” data using leave one out schemes, which is simpler to explain, and GCV which gives an approximation of CV. Despite the similarity in appearance between GCV and GML functions, the GML is computationally more expensive but sometimes give better result than GCV. This method is implemented in the *gss* package and ssanova() function in R.

For our problem, GML works well in some cases, as compared to GCV. However, GML can oversmooth the functions in many other cases. A few investigators [33] performed extensive simulation studies and concluded that the GCV and GML methods gave mixed results (one method was not superior to another in all situations).

For our data, we use the B-spline representation [39, p. 28]

$$\hat{f}_{i}\left( x_{it} \right)= \sum_{k=1}^{K} \hat{c}_{ik}\phi_{k}\left( x_{it} \right)$$

and choose K. Here, K is the number of basis, with K=number of knots + 2 [39]. We will first determine K by “myopic algorithm” as suggested in [34] then select $\lambda_{i}$ (with GCV). Quoting [34], the “algorithm for selecting the number of knots is as follows. First, the P-spline fit is computed for K equal to 5 and 10. In each case (a parameter) is chosen to minimize GCV for that number of knots. If GCV at K = 10 is greater than .98 times GCV at K = 5, then one concludes that further increases in K are unlikely to decrease GCV and one uses K = 5 or 10, whichever has the smallest GCV. Otherwise, one computes the P-spline fit with K = 20 and compares GCV for K = 10 with GCV for K = 20 in the same way one compared GCV for K = 5 and 10. One stops and uses K = 10 or 20 (whichever gives the smaller GCV) if GCV at K = 20 exceeds .98 times GCV at K = 10. Otherwise, one computes the P-spline at K = 40, and so on. The algorithm is called “ myopic” since it never looks beyond the value of K where it stops.” [34]. Please note that, although the algorithm is presented in terms of P-splines, we may extend it to the B-spline basis since the “methodology in this article for selecting the number of knots is applicable to other bases, for example, B-splines” [34, p. 739], and we can apply it to K whether it is the number of knots or the number of basis (since the difference is always 2 in our problem).

For our problem, K=40 is chosen. The graphical results comparing the smoothing parameter selection algorithm are given here:

We see that GCV tracks the data (defeating the purpose of smoothing), while GML can sometime be too smooth. Hence, the B-spline smoothing with K=40 works well with our data.

**C. Mathematical Details of FPCA**

Before we introduce the FPCA, we recall the PCA in the multivariate statistical analysis. The first step involves computing the eigenvalues and eigenvectors of sample covariance matrix, given by

$$S= \frac{1}{n-1}\sum_{i=1}^{n} (y_{i}-\bar{y})(y_{i}-\bar{y})'$$

where $y_{i}=(y_{i1},\ldots, y_{ip})$ is a p-dimensional vector of data and “prime” indicates a transpose of a matrix, and

$$\bar{y}=\frac{1}{n}\sum_{i=1}^{n} y_{i}$$

the sample mean vector. Hence S will be a $p\times p$ matrix with variances on its diagonal and covariances on off-diagonals [20].

The eigenvalues and eigenvectors are then computed from the equation

$$S\xi= \mu\xi(C.1)$$

where S is a sample covariance matrix, $\mu$ is the eigenvalue and $\xi$ is the eigenvector [20]. One then finds a sequence of eigenvalues and eigenvectors $\mu_{1},\ldots.,\mu_{p}$ and $\xi_{1}, \ldots,\xi_{p}$, respectively, by maximizing $\xi'S\xi$ subject to $\xi^{'}\xi=1$ [15]. These sequences can easily be computed using most scientific software (including R). Once we obtain the eigenvalues and eigenvectors, the multivariate PCA then uses them to establish directions of maximal variance but only needs a few components (relative to the original dimension p). One may also obtain the principal component score by taking an inner product of eigenvector and data, $\xi^{'}y$. The eigenvectors themselves can be useful in PCA but are more useful in FPCA, as we shall show.

Here is the mathematical description of the FPCA [15]. Similar to the usual PCA, we need to calculate the eigenvalues and eigenvectors of the sample covariance matrix. However, since we have smoothed data rather than a vector of numbers, the calculation of FPCA has some differences. For the functional data FPCA, instead of the sample covariance function S in the multivariate PCA, we construct a covariance function

$$\hat{v}\left( s,t \right)=\frac{1}{n-1}\sum_{i=1}^{n} \left[ \hat{f}_{i}\left( s \right)- \bar{f}\left( s \right) \right]\left[ \hat{f}_{i}\left( t \right)- \bar{f}\left( t \right) \right] (C.2)$$

where

$$\bar{f}\left( x \right)= \frac{1}{n}\sum_{i=1}^{n} \hat{f}_{i}\left( x \right)$$

and we have $\hat{f}_{i}\left( x \right)$ from the basis expansion Eq. (3). From these, we may represent Eq. (C.2) as

$$\hat{v}(s,t)=\frac{1}{n-1}\phi(s)'C'C\phi(t)$$

where $C=\{c_{ik}\}$ is an n by K matrix composed of the coefficients, and $\phi\left( x \right)=(\phi_{1}\left( x \right),\ldots,\phi_{K}\left( x \right))'$ is a K-dimensional vector of the (B-spline) basis functions. Furthermore, by setting the equation similar to Eq. (C.1), $S\xi= \mu\xi$, we can get

$$\int\hat{v}(s,t) \xi\left( t \right)dt= \mu\xi(s)$$

Here, $\mu$ is still the eigenvalue but $\xi(s)$ is now called the eigenfunction (rather than the eigenvector). It will be difficult to solve this equation directly, but there is an alternative way of solving this cleverly. It turns out that the eigenfunction also has the basis expansion

$$\xi\left( s \right)= \sum_{k=1}^{K} b_{k}\phi_{k}(s)=\phi\left( s \right)^{'}b$$

where $b_{k}$is some coefficient but the $\phi_{k}$ is the same basis as before! Hence, the problem is reduced to solving for $b_{k}$, which is explained in detail in Ramsay and Silverman [15, pp. 161-163]. This approach is quite different from that of multivariate PCA. In computing the functional PCA, we take full advantage of the basis functions.

For the FPCA, we need to determine a set of the eigenfunctions $\xi_{1}\left( x \right), \ldots,\xi_{K}\left( x \right)$and the corresponding eigenvalues $\mu_{1},\ldots.,\mu_{K}$ as well. Similarly to PCA, this achieved by maximizing

$$< \xi,V\xi> = \iint\hat{v}\left( s,t \right)\xi\left( t \right)dt \xi\left( s \right)ds= \mu\int\xi\left( s \right)\xi\left( s \right)ds$$

subject to$\int\xi_{h}\left( s \right)\xi_{h}\left( s \right) ds=1$ and $\int\xi_{h}\left( s \right)\xi_{l}\left( s \right) ds=0$ for $h\neq l$. To actually compute the component $\xi_{h}\left( x \right)$ (and the corresponding eigenvalue $\mu_{h}$), we can either discretize the functions to make everything into vectors and matrices or we can make use of the basis functions. We will follow the basis function approach, which is explained in Section 8.4 of [15] and implemented in the *fda* software package in R [39]. This process is repeated until we find all $\xi_{1}\left( x \right), \ldots,\xi_{H}\left( x \right)$, and we also get the corresponding eigenvalues $\mu_{1},\ldots.,\mu_{H}$ as well. This has the effect of maximizing the variance of the individual component $\xi_{h}\left( x \right)$ but yet will be orthogonal to the any other components that we determine. Also, by construction the first component (and the corresponding eigenvalue) will capture the most amount of variability in the data, the second component will have the second most variability in the data, and so on. The eigenfunctions $\xi_{1}\left( x \right), \ldots,\xi_{H}\left( x \right)$are the principal components (or harmonics) in the FPCA. We will select a few principal components to represent the most variation of the data, and we will also need to rotate the components to obtain the better interpretation.

When we rotate the principal components, we transform the eigenfunctions to make them more interpretable but yet preserve the orthogonality properties (we have the orthogonal transformation which preserves the mathematical properties of the original components). For this task, VARIMAX rotation is used [15], which is defined as a transformation $\psi=T\xi$ with $\xi=(\xi_{1}\left( x \right), \ldots,\xi_{H}\left( x \right))$ and T is an H$\times$H matrix, so that we obtain the transformed components$\psi=\left( \psi_{1}\left( x \right), \ldots,\psi_{H}\left( x \right) \right)$ [39]. The purpose of the VARIMAX is to maximize the variance again when the components are transformed (rotated). Once the components are rotated, the percent variability of the components changes as well.

Now, we define the functional principal component scores,

$$z_{hi}=\int\xi_{h}(t)\left[ \hat{f}_{i}\left( t \right)- \bar{f}\left( t \right) \right]dt$$

These give us a numerical summary of the component for each data point, and the points plotted on a plane (for h=2) allow us to compare the two groups.

Once we have rotated the components, we can create the VARIMAX rotated components (replace $\xi_{h}$ by $\psi_{h}$ in Eq. (4)) and hence obtain the rotated component scores (harmonics) as

$$z_{hi}^{*}=\int\psi_{h}(t)\left[ \hat{f}_{i}\left( t \right)- \bar{f}\left( t \right) \right]dt$$

**D. Details on the Classification Algorithms**

Here we briefly describe the algorithms used in this study.

**Logistic Regression (Logistic)**

Logistic regression is a simple method that can be used to predict the outcome of the input variables [40]. If we denote $x=(x_{1},\ldots, x_{p})$ as the input variables and y as the response (say, y=0 non-obese, y=1 obese), then we have that

$$ln\left( \frac{P(y=1|x)}{1-P(y=1|x)} \right)=x^{'}\beta+\beta_{0}$$

or

$$P\left( y=1 | x \right)= \frac{1}{1+e^{-{(x}^{'}\beta+\beta_{0})}}$$

which we interpret as the probability of obese (y=1) given the data x. If we have the full data y and x, any software fitting a logistic regression will give coefficient values β. Then we only need the input values x to determine $P\left( y=1 | x \right)$, which will be between zero and one, inclusive. Given the input x of an individual, we classify the individual as obese if $P\left( y=1 | x \right) > c$ where c is a cutoff, typically set at 0.5. Note that it is linear in terms of parameters, and it will not fit any data with a large number of input variables (p) (where p>n). In addition, if the input data follow exactly the pattern of the outcome variable, we have the so-called “complete separation” problem. However, some of these shortcomings can easily be overcome with simple adjustments, and the logistic regression is a popular method because of its simplicity.

**Support Vector Machine (SVM)**

Support vector machine (SVM) is a machine learning method for binary classification [41]. The concept of linear separating hyperplane $g\left( x \right)= x^{'}\beta+\beta_{0}$ is used to classify the points in p-dimension into two groups. The SVM transforms nonlinear classification into a simpler linear classification problem, using a kernel function $K(x,x^{*})$, with the separating hyperplane

$$g\left( x \right)=\sum_{i=1}^{n} \alpha_{i}y_{i}K\left( x,x_{i} \right)+\beta_{0}$$

and the classification criteria sign$[f\left( x \right)]$. The optimization criterion is that we maximize the margin (support vector) of the separating hyperplane to obtain the optimal separation, where margin is defined as $M=1/\left\| \beta\right\|$. The typical choice for the kernel is a radial basis (Gaussian) kernel,

$$K(x,x^{*}) = exp(-\gamma{\parallel x-x^{*}\parallel}^{2})$$

which is the default for most SVM software. Other kernels such as the polynomial kernel $K\left( x,x^{*} \right)=\left( 1+\left\langle x,x^{*} \right\rangle\right)^{d}$ or the neural network (hyperbolic tangent) kernel $K\left( x,x^{*} \right)=tanh(\kappa_{1}\left\langle x,x^{*} \right\rangle+\kappa_{2})$ may be used, but the Gaussian kernel is the most popular because of its similarities with and therefore its desirable properties from the Gaussian distribution. As the SVM method involves nonlinear kernel and optimization, it can be very computationally intensive as compared to logistic regression, but the SVM is designed for a large number of input variables (p>n), which logistic regression cannot handle.

**Random Forest (RF)**

The random forest (RF) is a method based on classification tree [42]. The classification tree method looks for the best classification of data by splitting each variable recursively and finding the optimal combination [43]. In other words, if we are given the data y and $x=(x_{1},\ldots, x_{p})$, the classification tree looks for the best split points $(t_{1},\ldots, t_{p})$ that gives us the decision rule. For example, if we have three input variables $x_{1}, x_{2},x_{3}$ each taking values between 0 and 10, then the classification tree algorithm may provide the split points $t_{1}=5, t_{2}=8, t_{3}=4.5$ such that we declare an input ${(x}_{1}, x_{2},x_{3})$ as obese if $x_{1}\geq5, x_{2}\leq8,x_{3}\geq4.5$, and non-obese otherwise. The determination of split points largely depends on algorithms for which we have many choices. Nevertheless, we see that it is easy to understand conceptually and is a popular method for classification. There are many refinements of tree method such as AdaBoost[44], but we consider the RF here, which consistently outperforms other tree-based methods. The RF involves building of the trees based on the technique of bootstrap [45]. The bootstrap method requires simply sampling the data with replacement, and if this is repeated many times, we obtain bootstrap samples and an ensemble of trees from the samples. The final classifier we take a majority vote among all the trees [32]. Because the RF is a tree-based method that involves recursively partitioning all variables to find the optimal splits and performing bootstrap resampling, the computational burden and runtime will be much greater compared to its competitors.

**Variable Reduction by Elastic Net**

Recently, the variable selection and penalized methods such as elastic net [47] has gained popularity in high-dimensional statistical problems. We have also attempted to apply the elastic net, where the least absolute shrinkage and selection operator (LASSO) is a special case

(alpha=1), described in [47] and implemented by glmnet function in R. We obtain results comparable to that of FPC-based results. For example, we have considered the glmnet for the logistic regression. First, we considered LASSO with all the data, with the graphical result:

As we can see, the best subset (with 27 components) has the misclassification rate around 63 percent, which is comparable to the results given by FPCA in the paper.

We have then fit the elastic net with alpha=1/2.

Here, the results are worse, and other adjustment (different values of alpha) did not improve the results. One may try to find the “best” alpha (by CV or other parameter search/selection methods), but here the LASSO (alpha=1) gives the best result, and in general it will place additional burden on the users.

Since each points in the domain are time, it is difficult to interpret which times were selected by LASSO or elastic net. Moreover, the data is functional, meaning that there are correlations over time, and FPCA is better suited to handle such data. The elastic net is more suitable for high dimensional data (such as gene expression). Overall, the interpretation become more difficult with glmnet, because it selects time variables at disparate times while our data has time variables that are correlated. Hence, it is more sensible to consider FPCA in our problem.

We have also tried fitting LASSO to functional principal components to see which ones it selects and if there is any improvement. The graphical results are below

We see that LASSO only picks one component (the first component) according to CV, and the results are again not much improved from FPC alone. If we relax the selection conditions, it picks up components that are far down the line (with corresponding eigenvalue near zero), making the interpretation more difficult again but not improving the result. The result with elastic net and other variations of glmnet give similar results. Therefore, using FPCA with simple classifier seems to work the best in our situation.
